# Dupilumab-Associated Mycosis Fungoides with a CD8+ Immunophenotype

**DOI:** 10.3390/dermatopathology9040045

**Published:** 2022-11-30

**Authors:** Ariel Park, Lulu Wong, Annalise Lang, Christina Kraus, Nancy Anderson, Ashley Elsensohn

**Affiliations:** 1 Department of Dermatology, Loma Linda University, Loma Linda, CA 92354, USA; 2 Department of Dermatology, University of California, Irvine, CA 92697, USA

**Keywords:** CD8+, cutaneous T-cell lymphoma, CTCL, dupilumab, mycosis fungoides

## Abstract

Dupilumab is a humanized IgG4 monoclonal-antibody that is approved by the United States Food and Drug Administration (FDA) for the treatment of moderate-to-severe atopic dermatitis (AD) in patients aged 12 years and older. In recent years, several case studies have associated the unmasking or progression of cutaneous T-cell lymphomas (CTCL) with dupilumab treatment. To date, all reported cases of dupilumab-associated CTCL have shown a CD4+ T-helper-cell-predominant immunophenotype. Here, we report a case of a 72-year-old man who presented with a 2-year history of a diffuse, pruritic eruption, who was started on dupilumab for 9 weeks. He subsequently developed mycosis fungoides (MF) with a CD8+-predominant immunophenotype. Overall, cases of CD8+ mycosis fungoides are less common and relatively less understood than their CD4+ counterparts, with varied presentations and courses. We present a case of dupilumab-associated CD8+ MF to highlight this presentation for pathologists and providers.

## 1. Introduction

Dupilumab is a humanized IgG4 monoclonal antibody that targets the IL-4 receptor alpha chain in the heterodimeric IL-4 receptor complex, blocking signaling of IL-4 and IL-13. These type 2 cytokines are known to play a key role in the pathogenesis of allergic disorders such as atopic dermatitis (AD), asthma, and food allergies [1]. In 2017, dupilumab was approved by the United States Food and Drug Administration (FDA) for the treatment of moderate-to-severe AD in adults uncontrolled with topical medications, becoming the first biologic agent available to treat AD [1]. Several case studies in recent years have associated unmasking or progression of cutaneous T-cell lymphomas (CTCL) with dupilumab treatment [2,3,4,5,6,7,8,9,10,11,12,13]. To date, all cases that have reported a CD4:CD8 ratio have shown a greater proportion of CD4+ cells [14]. Here we highlight a case of dupilumab-associated mycosis fungoides (MF) with a CD8+ predominant immunophenotype.

## 2. Case Synopsis

**Figure 1:**
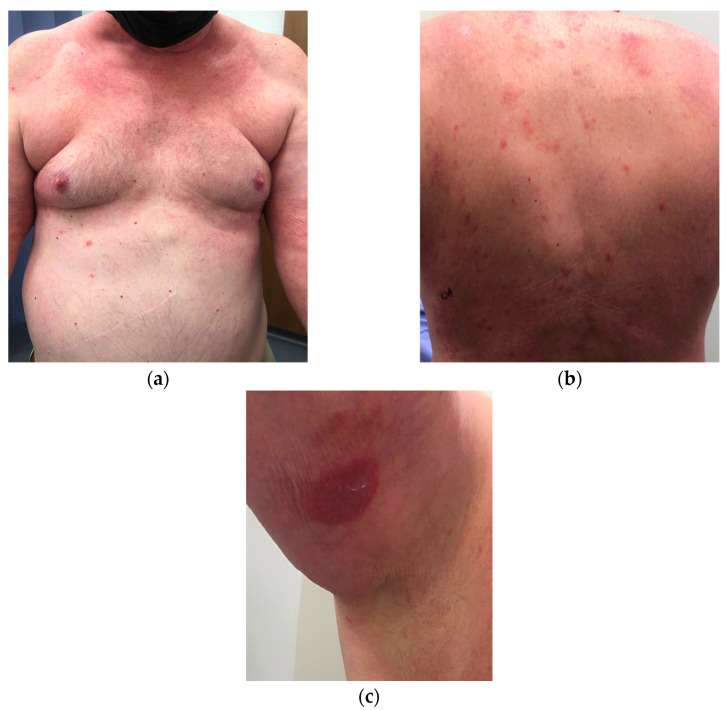
Clinical appearance of erythematous, poorly demarcated, faintly scaly plaques on the: (**a**) trunk; (**b**) and back. (**c**) A circular erythematous scaly-plaque on the upper-right inner arm.

A 72-year-old male presented to the dermatology clinic with a diffuse, pruritic eruption of 2-years duration that started in the groin and progressed to involve the trunk, extremities, and face. The patient’s medical history was significant for hypertension, diabetes, chronic kidney disease, and exposure to Agent Orange. Prior biopsies from an outside dermatologist showed lichenoid interface and spongiotic patterns. The patient did not improve with clobetasol, fluocinonide, tacrolimus, and topical antifungals. Dupilumab was then started, with progression of his dermatitis over 9 weeks. Upon presentation to our clinic, physical examination revealed erythematous, poorly demarcated, faintly scaly plaques on the trunk, a confluent erythematous, non-scaly plaque on the scrotum, and a round erythematous scaly plaque on the right inner arm. There was also diffuse erythema on the neck and upper chest. The total body-surface-area involved was approximately 12–15%. Biopsies were performed on two sites on the back and right arm (Figure 1). Dupilumab was discontinued and clobetasol 0.05% ointment and light therapy were initiated.

**Figure 2:**
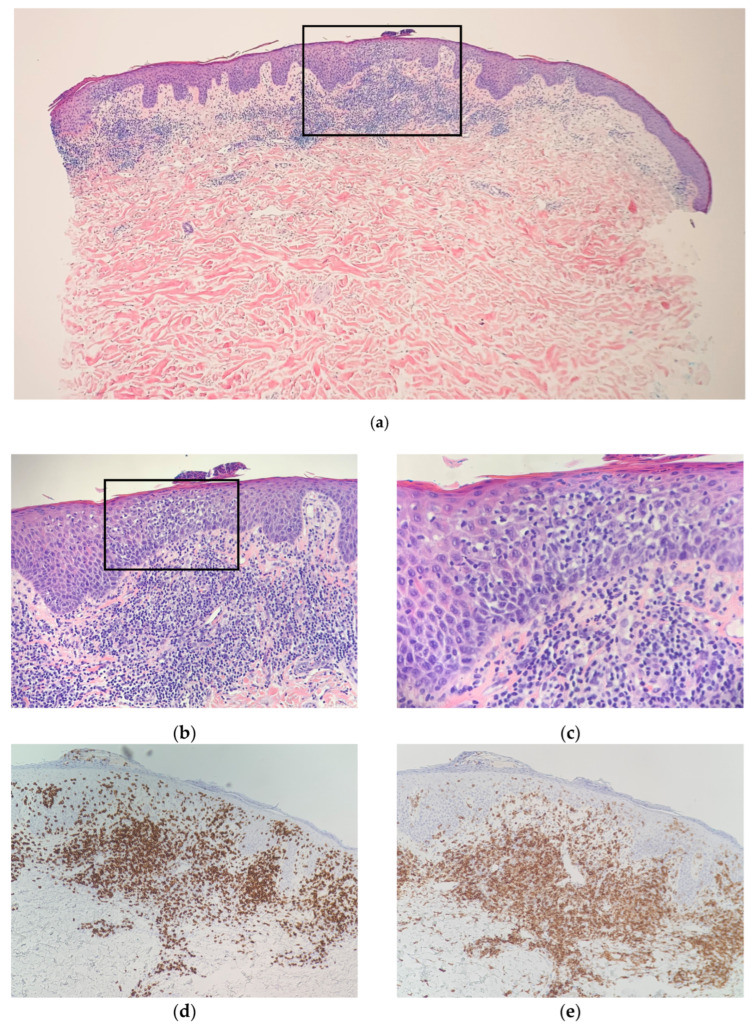
Left mid-back punch-biopsy histology (hematoxylin-eosin) showing interface dermatitis with hyperchromatic, angular, atypical lymphocytes in the epidermis and at the dermal–epidermal junction with accompanying papillary dermal-fibrosis at magnification (**a**) ×20; (**b**) ×40; (**c**) ×100; (**d**) Immunohistochemical staining of CD8, magnification ×40; (**e**) Immunohistochemical staining of CD4, magnification ×40. Consistent with CD8+-predominant mycosis fungoides (cutaneous T-cell lymphoma).

**Figure 3:**
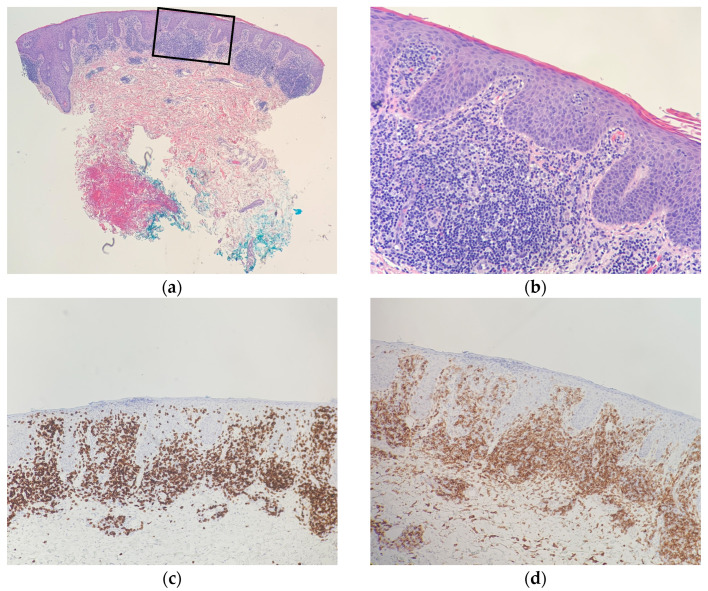
Right upper-arm punch-biopsy histology (hematoxylin-eosin) at magnification (**a**) ×20; (**b**) and ×40; (**c**) Immunohistochemical staining of CD8, magnification ×40; (**d**) Immunohistochemical staining of CD4, magnification ×40. Consistent with CD8+-predominant mycosis fungoides (cutaneous T-cell lymphoma).

A histopathologic exam showed an interface dermatitis with hyperchromatic, angular, atypical lymphocytes in the epidermis and at the dermal-epidermal junction, with accompanying papillary dermal fibrosis. Immunohistochemical stains were positive for CD3 (CD3 highlighted numerous T cells in the epidermis) and showed a predominance of CD8+ over CD4+ expression with preservation of CD5 (Figure 2 and Figure 3). The TCR-gene rearrangement studies were negative. The overall histologic findings, in conjunction with the clinical picture, supported a diagnosis of CD8+ mycosis fungoides. The patient was continued on topical and light therapy and referred to Hematology-Oncology for adjunctive management. Flow cytometry, bone marrow biopsy, and positron emission tomography (PET)/computed tomography (CT) were within normal limits. The patient’s CTCL was staged as mycosis fungoides stage IIA. The patient was started on extracorporeal photopheresis twice a week and bexarotene 75 mg daily. In subsequent months, the patient was seen at an NCI-designated cancer-referral center. The histopathology was again reviewed. Additional immunophenotyping was carried out, which showed partial loss of CD2, loss of CD7, beta F1 expression, and CD56 negativity. Altogether, the findings were thought to be consistent with CD8+-predominant mycosis fungoides. Several months into therapy, the patient has not progressed in his disease.

## 3. Discussion

In recent years, several case studies have associated the unmasking or progression of CTCL with dupilumab treatment [2,3,4,5,6,7,8,9,10,11,12,13]. Dupilumab competitively inhibits IL-4 and IL-13 at the IL-4 α1 subunit receptor, preventing the downstream activation of tyrosine kinases promoting gene-transcription and related to both barrier dysfunction and Th2-mediated inflammation [15]. The IL-13 upregulation has been associated with the pathogenesis of several malignancies, including cutaneous lymphoma, and it was thought that, theoretically, suppression of IL-13 may be beneficial for CTCL. However, this has not been the case in clinical practice, with some patients developing rapidly progressive cutaneous-lymphoma while on dupilumab, suggesting a more complex relationship between the IL-4-receptor blockade and cutaneous lymphoma [16]. In addition to the IL-4 receptor, IL-13 binds to a lesser-known receptor, the IL-13 α2 subunit receptor (IL-13Rα2), of which the function remains unclear. It has been postulated that this receptor acts as a “decoy” by binding to the cytokine without downstream effects, reducing IL-13 levels in the serum and further inhibiting the IL-4 pathway [17]. However, the downstream effects of IL-13Rα2 may act as more than just a “decoy”, with updated evidence suggesting an association with a worse prognosis and a potential role in cell proliferation and invasion, and immune evasion of various cancers [18,19,20,21]. Despite these various hypotheses, the mechanism by which dupilumab-associated CTCL occurs is not fully known.

A study by Sokumbi et al. discussed the evolution of histopathologic and immunophenotypic features of lymphoid infiltrates in seven cases of CTCL after treatment of biopsy-proven AD with dupilumab [11]. Due to the progressive increase in the density of atypical lymphoid cells, prominent collagen fibrosis in the papillary dermis, and epidermotropic lymphocytes across pre-dupilumab and post-dupilumab biopsies, the authors recommend serial biopsies before and during treatment with dupilumab [11]. In addition, initial biopsy specimens may resemble inflammatory skin-diseases, due to a predominance of reactive lymphocytes and lack of cytologically atypical lymphocytes, leading to low concordance-rates among pathologists for the diagnosis for early MF, further emphasizing the need for serial biopsies from various clinical sites for a definitive diagnosis [22].

To date, 12 studies including a total of 27 patients report the development of CTCL after dupilumab treatment for an initial diagnosis other than CTCL, or demonstrate exacerbation of CTCL with dupilumab [14]. Our case demonstrates such a conundrum, where several outside biopsies were performed showing spongiotic and lichenoid patterns. Whether these prior biopsies were undiagnosed “early” CTCL, or the dupilumab did in fact induce or exacerbate CTCL, is an area for further exploration. Of the 14 cases that include the CD4:CD8 ratio, all had a greater proportion of CD4 cells than CD8 cells [6,8,11,12,13]. Eight of these studies showed a CD4:CD8 ratio of ≥20:1 [6,11,12].

For a complete review of demographic and histologic findings of recently reported dupilumab-associated CTCL cases, please refer to the systematic review [14].

The most common subtype of CTCL is MF, but only about 5% of MF cases are CD8-positive [23]. The understanding of CD8+ CTCL is still limited. Massone et al. evaluated 73 specimens from 68 patients with early-stage disease at diagnosis (either stage Ia or Ib), and suggested that at early stages, CD8+ MF does not behave differently to CD4+ MF, and does not necessarily have a worse prognosis [24]. However, another study highlighted the fact that CD8+ MF is not a single entity, but rather a ‘mixed-bag’ of presentations, with some having more indolent courses similar to the typical CD4+ MF, such as those with hypopigmented patches often found in the younger population, and others demonstrating a more aggressive course [25,26]. Other studies comparing CD8+- and CD4+-predominant MF cohorts, suggest that CD8+ MF has a more indolent course, and suggest a treatment approach limited to skin-directed therapies and observation, for most patients [26,27].

Treatment options and outcomes for patients with dupilumab-associated CTCL are varied. Discontinuation of dupilumab upon CTCL diagnosis is usually recommended. However, there have been paradoxical reports of clinical improvement in CTCL, especially in pruritus, with dupilumab use [6,28].

## 4. Conclusions

Our case contributes to the growing literature and understanding of CD8+-predominant CTCL and is the first to describe CD8+-predominant mycosis fungoides associated with dupilumab use. Awareness of the varied and distinct entities of MF and repeat histopathological-evaluation in patients suspected of having MF, or who have progressive disease-severity, on dupilumab, is important to avoid a delayed diagnosis. Future studies should focus on identifying whether prognosis and course differ for the different immunophenotypes of dupilumab-associated MF. Additionally, further studies are needed to evaluate optimal treatment-modalities for patients with dupilumab-associated MF.
